# Tetra­aqua­bis(nicotinamide-κ*N* ^1^)cobalt(II) bis­(2-fluoro­benzoate)

**DOI:** 10.1107/S1600536809006771

**Published:** 2009-02-28

**Authors:** F. Elif Özbek, Barış Tercan, Ertan Şahin, Hacali Necefoğlu, Tuncer Hökelek

**Affiliations:** a Department of Chemistry, Kafkas University, 63100 Kars, Turkey; b Department of Physics, Karabük University, 78050, Karabük, Turkey; c Department of Chemistry, Atatürk University, 22240 Erzurum, Turkey; d Department of Physics, Hacettepe University, 06800 Beytepe, Ankara, Turkey

## Abstract

The title complex, [Co(C_6_H_6_N_2_O)_2_(H_2_O)_4_](C_7_H_4_FO_2_)_2_, contains one Co(II) atom (site symmetry 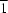), two monodentate nicotin­amide (NA) ligands, four coordinated water mol­ecules and two 2-fluoro­benzoate (FB) anions. The four O atoms in the equatorial plane around the Co atom form a slightly distorted square-planar arrangement, while the slightly distorted octa­hedral coordination is completed by the two N atoms of the NA ligands in the axial positions. The dihedral angle between the carboxyl group and the adjacent benzene ring is 29.8 (3)°, while the pyridine and benzene rings are oriented at a dihedral angle of 7.97 (12)°. In the crystal structure, mol­ecules are linked by O—H⋯O, N—H⋯O and N—H⋯F hydrogen bonds, forming an infinite three-dimensional network. π–π Contacts between the pyridine and benzene rings [centroid–centroid distance = 3.673 (3) Å] may further stabilize the crystal structure.

## Related literature

For general background, see: Antolini *et al.* (1982 ▶); Krishnamachari (1974 ▶); Nadzhafov *et al.* (1981 ▶). For related structures, see: Hökelek & Necefoğlu (1996 ▶, 1998 ▶); Hökelek *et al.* (1997 ▶, 2007 ▶); Necefoğlu *et al.* (2002 ▶); Tercan *et al.* (2009 ▶).
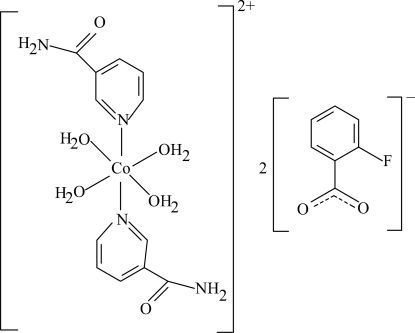


## Experimental

### 

#### Crystal data

[Co(C_6_H_6_N_2_O)_2_(H_2_O)_4_](C_7_H_4_FO_2_)_2_
*M* *_r_* = 653.45
Triclinic, 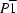

*a* = 7.2913 (2) Å
*b* = 7.4522 (4) Å
*c* = 14.4853 (5) Å
α = 82.160 (2)°
β = 77.275 (3)°
γ = 63.740 (3)°
*V* = 687.83 (5) Å^3^
*Z* = 1
Mo *K*α radiation
μ = 0.70 mm^−1^
*T* = 294 K
0.35 × 0.25 × 0.20 mm

#### Data collection

Rigaku R-AXIS RAPID-S diffractometer
Absorption correction: multi-scan (Blessing, 1995 ▶) *T*_min_ = 0.807, *T*_max_ = 0.865
14875 measured reflections
2817 independent reflections
2679 reflections with *I* > 2σ(*I*)
*R*_int_ = 0.044

#### Refinement

*R*[*F* ^2^ > 2σ(*F* ^2^)] = 0.042
*wR*(*F* ^2^) = 0.122
*S* = 1.08
2817 reflections
220 parameters
10 restraints
H atoms treated by a mixture of independent and constrained refinement
Δρ_max_ = 1.37 e Å^−3^
Δρ_min_ = −0.42 e Å^−3^

### 

Data collection: *CrystalClear* (Rigaku/MSC, 2005 ▶); cell refinement: *CrystalClear*; data reduction: *CrystalClear*; program(s) used to solve structure: *SHELXS97* (Sheldrick, 2008 ▶); program(s) used to refine structure: *SHELXL97* (Sheldrick, 2008 ▶); molecular graphics: *Mercury* (Macrae *et al.*, 2006 ▶); software used to prepare material for publication: *WinGX* (Farrugia, 1999 ▶).

## Supplementary Material

Crystal structure: contains datablocks I, global. DOI: 10.1107/S1600536809006771/xu2482sup1.cif

Structure factors: contains datablocks I. DOI: 10.1107/S1600536809006771/xu2482Isup2.hkl

Additional supplementary materials:  crystallographic information; 3D view; checkCIF report

## Figures and Tables

**Table:** 

Co1—O4	2.143 (3)
Co1—O5	2.075 (3)
Co1—N1	2.145 (3)

**Table:** 

O4—Co1—N1	93.69 (10)
O4—Co1—N1^i^	86.31 (10)
O5^i^—Co1—N1	92.59 (11)
O5—Co1—N1	87.41 (11)

Symmetry code: (i) 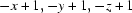.

**Table 2:** Hydrogen-bond geometry (Å, °)

*D*—H⋯*A*	*D*—H	H⋯*A*	*D*⋯*A*	*D*—H⋯*A*
N2—H21⋯O1^ii^	0.87 (2)	2.04 (3)	2.902 (5)	171 (4)
N2—H22⋯F1^iii^	0.87 (2)	2.54 (4)	2.916 (5)	107 (2)
N2—H22⋯O2^iii^	0.87 (2)	2.26 (3)	3.116 (5)	172 (4)
O4—H41⋯O3	0.91 (5)	2.06 (4)	2.885 (4)	151 (4)
O4—H42⋯O3^iv^	0.90 (3)	1.86 (5)	2.761 (4)	178 (5)
O5—H51⋯O2^v^	0.90 (4)	1.80 (4)	2.695 (4)	172 (4)
O5—H52⋯O3^vi^	0.91 (2)	1.95 (4)	2.798 (4)	156 (4)

Symmetry codes: (ii) 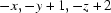; (iii) 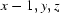; (iv) 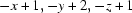; (v) 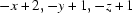; (vi) 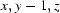.

**Fig. 1.**
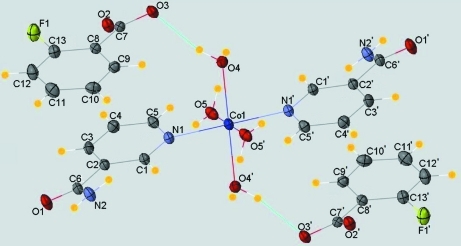
The molecular structure of the title molecule with the atom-numbering scheme. Displacement ellipsoids are drawn at the 40% probability level [symmetry code ('): -x, -y, -z]. Hydrogen bonds are shown as dotted lines.

**Fig. 2.**
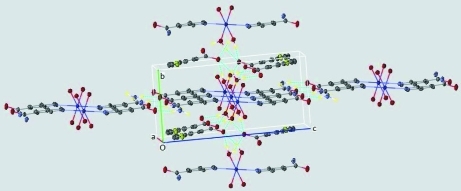
A partial packing diagram of the title compound. Hydrogen bonds are shown as dotted lines. H atoms not involved in hydrogen bonding are omitted.

## supplementary crystallographic information

### Comment

Transition metal complexes with biochemically active ligands frequently show interesting physical and/or chemical properties, through which they may find applications in biological systems (Antolini *et al.*, 1982). The structural functions and coordination relationships of the arylcarboxylate ion in transition metal complexes of benzoic acid derivatives may be changed, depending on the nature and position of the substituted groups on the benzene ring, the nature of the additional ligand molecule or solvent, and the medium of the synthesis (Nadzhafov *et al.*, 1981). Nicotinamide (NA) is one form of niacin and a deficiency of this vitamin leads to loss of copper from the body, known as pellagra disease. Victims of pellagra show unusually high serum and urinary copper levels (Krishnamachari, 1974). The structure determination of the title compound, (I), a cobalt complex with two nicotinamide (NA) ligands, four water molecules and two 2-fluorobenzoate (FB) anions, was undertaken in order to determine the properties of the NA ligands and FB anions and also to compare the results obtained with those reported previously.

Compound (I) is a monomeric complex, with the Co atom on a centre of symmetry. It contains two NA ligands, four water molecules and two FB molecules (Fig. 1). The NA ligands are monodentate. The four O atoms (O4, O5, and the symmetry-related atoms, O4', O5') in the equatorial plane around the Co atom form a slightly distorted square-planar arrangement, while the slightly distorted octahedral coordination is completed by the two N atoms of the NA ligands (N1, N1') in the axial positions (Table 1 and Fig. 1). The intramolecular O—H···O hydrogen bonds (Table 2) link two of the water molecules to the two FB anions.

The near equality of the C7—O2 [1.244 (4) Å] and C7—O3 [1.270 (4) Å] bonds in the carboxylate group indicates a delocalized bonding arrangement, rather than localized single and double bonds, and may be compared with the corresponding distances: 1.279 (4) and 1.246 (4) Å in bis(µ-4-hydroxybenzoato-*O*:*O*')bis- [(*N*,*N*-diethylnicotinamide-*N*^1^)-(4-hydroxybenzoato-O)zinc(II)] dihydrate (Hökelek & Necefouglu, 1996), 1.267 (3) and 1.237 (4) Å in *trans*-diaquabis- (*N*,*N*-diethylnicotinamide-*N*^1^)bis(4-nitrobenzoato-O)copper(II) (Hökelek *et al.*, 1997), 1.254 (2) and 1.251 (2) Å in *trans*-diaquabis(nicotinamide-N^1^)- bis(4-nitrobenzoato-O)cobalt(II) (Hökelek & Necefouglu, 1998), 1.240 (3), 1.281 (3) and 1.274 (3), 1.245 (3) Å in bis(4-hydroxybenzoato-κ*O*)bis(nicotin amide-κ*N*)zinc(II) (Necefoğlu *et al.*, 2002), 1.260 (4) and 1.252 (4) Å in diaquabis(*N*,*N*'-diethylnicotinamide-κ*N*)bis(4-fluorobenzoato-κ*O*)zinc(II) (Hökelek *et al.*, 2007) and 1.284 (2), 1.248 (2) and 1.278 (2), 1.241 (2) Å in bis[4-(methylamino)benzoato-κ*O*]bis(nicotinamide-κ*N*)zinc(II) (Tercan *et al.*, 2009). This may be due to the intramolecular O—H···O hydrogen bonding of the carboxylate O atom (Table 2).

The dihedral angle between the planar carboxylate group (O2/C7/O3) and the adjacent benzene B (C8—C14) ring is 29.8 (3)°. The dihedral angle between the pyridine ring A (N1/C1—C5) and benzene ring B is 7.97 (12)°.

As can be seen from the packing diagram (Fig. 2), the molecules are linked by O—H···O, N—H···O and N—H···F hydrogen bonds (Table 2) to form an infinite three-dimensional network, in which they may be effective in the stabilization of the structure. The π-π contact between the pyridine and the benzene rings, *Cg*1—*Cg*2 [where *Cg*1 and *Cg*2 are centroids of the rings A (N1/C1—C5) and B (C8—C14), respectively] may further stabilize the structure, with centroid-centroid distance of 3.673 (3) Å.

### Experimental

The title compound was prepared by the reaction of CoSO_4_.7(H_2_O) (1.40 g, 5 mmol) in H_2_O (20 ml) and NA (1.22 g, 10 mmol) in H_2_O (20 ml) with 2-fluorobenzoate (1.62 g, 10 mmol) in H_2_O (50 ml) at room temperature. The mixture was filtered and set aside to crystallize at ambient temperature for one week, giving pink single crystals.

### Refinement

H atoms of water molecules and NH_2_ group were located in difference syntheses and refined isotropically [O—H = 0.90 (3)–0.91 (5) Å, *U*_iso_(H) = 0.053 (12) -0.10 (2) Å^2^; N—H = 0.87 (2) Å, *U*_iso_(H) = 0.047 (11) and 0.041 (10) Å^2^]. The remaining H atoms were positioned geometrically with C—H = 0.93 Å, for aromatic H atoms and constrained to ride on their parent atoms, with *U*_iso_(H) = 1.2*U*_eq_(C). The restrains on the N—H bonds and the H—N—H bond angles of the NH_2_ group and O—H bonds and H—O—H bond angles of water molecules were applied.

### Crystal data

**Table:**

[Co(C_6_H_6_N_2_O)_2_(H_2_O)_4_](C_7_H_4_FO_2_)_2_	*Z* = 1
*M**_r_* = 653.45	*F*(000) = 337
Triclinic, *P*1	*D*_x_ = 1.578 Mg m^−^^3^
Hall symbol: -P 1	Mo *K*α radiation, λ = 0.71073 Å
*a* = 7.2913 (2) Å	Cell parameters from 4045 reflections
*b* = 7.4522 (4) Å	θ = 2.9–26.4°
*c* = 14.4853 (5) Å	µ = 0.70 mm^−^^1^
α = 82.160 (2)°	*T* = 294 K
β = 77.275 (3)°	Prism, pink
γ = 63.740 (3)°	0.35 × 0.25 × 0.20 mm
*V* = 687.83 (5) Å^3^	

### Data collection

**Table:**

Rigaku R-AXIS RAPID-S diffractometer	2817 independent reflections
Radiation source: fine-focus sealed tube	2679 reflections with *I* > 2σ(*I*)
graphite	*R*_int_ = 0.044
ω scans	θ_max_ = 26.4°, θ_min_ = 2.9°
Absorption correction: multi-scan (Blessing, 1995)	*h* = −9→9
*T*_min_ = 0.807, *T*_max_ = 0.865	*k* = −8→9
14875 measured reflections	*l* = −18→18

### Refinement

**Table:**

Refinement on *F*^2^	Primary atom site location: structure-invariant direct methods
Least-squares matrix: full	Secondary atom site location: difference Fourier map
*R*[*F*^2^ > 2σ(*F*^2^)] = 0.042	Hydrogen site location: inferred from neighbouring sites
*wR*(*F*^2^) = 0.122	H atoms treated by a mixture of independent and constrained refinement
*S* = 1.08	*w* = 1/[σ^2^(*F*_o_^2^) + (0.0649*P*)^2^ + 0.4285*P*] where *P* = (*F*_o_^2^ + 2*F*_c_^2^)/3
2817 reflections	(Δ/σ)_max_ < 0.001
220 parameters	Δρ_max_ = 1.37 e Å^−^^3^
10 restraints	Δρ_min_ = −0.42 e Å^−^^3^

### Special details

**Table:**

Geometry. All e.s.d.'s (except the e.s.d. in the dihedral angle between two l.s. planes) are estimated using the full covariance matrix. The cell e.s.d.'s are taken into account individually in the estimation of e.s.d.'s in distances, angles and torsion angles; correlations between e.s.d.'s in cell parameters are only used when they are defined by crystal symmetry. An approximate (isotropic) treatment of cell e.s.d.'s is used for estimating e.s.d.'s involving l.s. planes.
Refinement. Refinement of *F*^2^ against ALL reflections. The weighted *R*-factor *wR* and goodness of fit *S* are based on *F*^2^, conventional *R*-factors *R* are based on *F*, with *F* set to zero for negative *F*^2^. The threshold expression of *F*^2^ > σ(*F*^2^) is used only for calculating *R*-factors(gt) *etc*. and is not relevant to the choice of reflections for refinement. *R*-factors based on *F*^2^ are statistically about twice as large as those based on *F*, and *R*- factors based on ALL data will be even larger.

### Fractional atomic coordinates and isotropic or equivalent isotropic displacement parameters (Å^2^)

**Table:**

	*x*	*y*	*z*	*U*_iso_*/*U*_eq_	
Co1	0.5000	0.5000	0.5000	0.0306 (2)	
F1	0.6929 (4)	0.9615 (4)	0.85873 (18)	0.0632 (7)	
O1	0.2777 (4)	0.4281 (5)	0.97121 (18)	0.0544 (7)	
O2	0.9068 (4)	0.8007 (4)	0.68460 (17)	0.0433 (6)	
O3	0.7033 (4)	0.9537 (4)	0.57755 (17)	0.0414 (6)	
O4	0.6387 (4)	0.7065 (4)	0.46282 (17)	0.0398 (6)	
H41	0.695 (6)	0.741 (6)	0.504 (3)	0.057 (13)*	
H42	0.529 (7)	0.818 (6)	0.449 (4)	0.10 (2)*	
O5	0.7856 (4)	0.2570 (4)	0.46679 (18)	0.0450 (6)	
H51	0.896 (5)	0.227 (7)	0.419 (3)	0.070 (15)*	
H52	0.799 (7)	0.140 (5)	0.499 (3)	0.053 (12)*	
N1	0.5506 (4)	0.4540 (4)	0.64340 (18)	0.0334 (6)	
N2	0.0471 (5)	0.5743 (5)	0.8720 (2)	0.0460 (8)	
H21	−0.056 (5)	0.588 (6)	0.918 (2)	0.047 (11)*	
H22	0.019 (6)	0.640 (5)	0.819 (2)	0.041 (10)*	
C1	0.3949 (5)	0.4742 (5)	0.7168 (2)	0.0337 (7)	
H1	0.2679	0.4926	0.7042	0.040*	
C2	0.4139 (5)	0.4691 (5)	0.8104 (2)	0.0348 (7)	
C3	0.6044 (6)	0.4392 (5)	0.8293 (2)	0.0393 (8)	
H3	0.6229	0.4340	0.8913	0.047*	
C4	0.7650 (5)	0.4175 (5)	0.7546 (3)	0.0408 (8)	
H4	0.8937	0.3981	0.7654	0.049*	
C5	0.7332 (5)	0.4248 (5)	0.6634 (2)	0.0380 (7)	
H5	0.8434	0.4089	0.6135	0.046*	
C6	0.2384 (6)	0.4906 (6)	0.8914 (2)	0.0393 (8)	
C7	0.7326 (5)	0.8934 (5)	0.6617 (2)	0.0326 (7)	
C8	0.5407 (5)	0.9340 (5)	0.7363 (2)	0.0330 (7)	
C9	0.3610 (5)	0.9450 (5)	0.7114 (3)	0.0381 (7)	
H9	0.3613	0.9304	0.6486	0.046*	
C10	0.1833 (6)	0.9768 (6)	0.7779 (3)	0.0474 (9)	
H10	0.0663	0.9813	0.7601	0.057*	
C11	0.1798 (7)	1.0021 (7)	0.8708 (3)	0.0572 (11)	
H11	0.0593	1.0261	0.9155	0.069*	
C12	0.3539 (7)	0.9920 (7)	0.8980 (3)	0.0544 (10)	
H12	0.3526	1.0070	0.9609	0.065*	
C13	0.5296 (6)	0.9593 (6)	0.8305 (2)	0.0411 (8)	

### Atomic displacement parameters (Å^2^)

**Table:**

	*U*^11^	*U*^22^	*U*^33^	*U*^12^	*U*^13^	*U*^23^
Co1	0.0292 (3)	0.0361 (4)	0.0231 (3)	−0.0124 (3)	−0.0019 (2)	−0.0010 (2)
F1	0.0543 (14)	0.0868 (19)	0.0507 (14)	−0.0276 (14)	−0.0144 (11)	−0.0109 (13)
O1	0.0510 (16)	0.084 (2)	0.0263 (13)	−0.0306 (15)	−0.0069 (11)	0.0080 (13)
O2	0.0319 (12)	0.0595 (16)	0.0341 (13)	−0.0166 (11)	−0.0066 (10)	0.0025 (11)
O3	0.0418 (13)	0.0504 (14)	0.0300 (12)	−0.0196 (11)	−0.0077 (10)	0.0065 (10)
O4	0.0449 (14)	0.0440 (14)	0.0350 (13)	−0.0226 (12)	−0.0102 (11)	0.0017 (10)
O5	0.0385 (14)	0.0435 (14)	0.0371 (14)	−0.0100 (11)	0.0063 (11)	0.0005 (11)
N1	0.0340 (14)	0.0389 (15)	0.0252 (13)	−0.0145 (12)	−0.0041 (10)	−0.0003 (11)
N2	0.0397 (17)	0.063 (2)	0.0298 (16)	−0.0209 (15)	−0.0032 (13)	0.0059 (14)
C1	0.0331 (16)	0.0400 (17)	0.0272 (15)	−0.0151 (14)	−0.0060 (12)	−0.0001 (13)
C2	0.0388 (17)	0.0374 (17)	0.0269 (15)	−0.0161 (14)	−0.0057 (13)	0.0014 (13)
C3	0.0453 (19)	0.0452 (19)	0.0297 (16)	−0.0196 (16)	−0.0137 (14)	0.0028 (14)
C4	0.0348 (17)	0.047 (2)	0.0424 (19)	−0.0175 (15)	−0.0128 (14)	0.0020 (15)
C5	0.0325 (16)	0.0429 (18)	0.0353 (17)	−0.0147 (14)	−0.0032 (13)	−0.0005 (14)
C6	0.048 (2)	0.048 (2)	0.0246 (16)	−0.0241 (17)	−0.0045 (14)	0.0009 (14)
C7	0.0362 (17)	0.0342 (16)	0.0306 (16)	−0.0177 (14)	−0.0068 (13)	−0.0004 (12)
C8	0.0335 (16)	0.0317 (16)	0.0322 (16)	−0.0137 (13)	−0.0052 (13)	0.0012 (12)
C9	0.0386 (18)	0.0392 (18)	0.0396 (18)	−0.0188 (15)	−0.0092 (14)	0.0000 (14)
C10	0.0323 (18)	0.050 (2)	0.060 (2)	−0.0193 (16)	−0.0063 (16)	0.0021 (18)
C11	0.042 (2)	0.068 (3)	0.052 (2)	−0.022 (2)	0.0101 (18)	−0.008 (2)
C12	0.051 (2)	0.070 (3)	0.035 (2)	−0.022 (2)	0.0032 (17)	−0.0094 (18)
C13	0.0383 (18)	0.047 (2)	0.0354 (18)	−0.0158 (16)	−0.0067 (14)	−0.0024 (15)

### Geometric parameters (Å, °)

**Table:**

Co1—O4^i^	2.143 (3)	C2—C1	1.387 (4)
Co1—O4	2.143 (3)	C2—C3	1.390 (5)
Co1—O5^i^	2.075 (3)	C3—H3	0.9300
Co1—O5	2.075 (3)	C4—C3	1.375 (5)
Co1—N1	2.145 (3)	C4—H4	0.9300
Co1—N1^i^	2.145 (3)	C5—C4	1.381 (5)
F1—C13	1.348 (4)	C5—H5	0.9300
O1—C6	1.232 (4)	C6—C2	1.499 (5)
O2—C7	1.244 (4)	C7—C8	1.505 (5)
O3—C7	1.270 (4)	C8—C9	1.399 (5)
O4—H41	0.91 (5)	C8—C13	1.384 (5)
O4—H42	0.90 (3)	C9—C10	1.379 (5)
O5—H51	0.90 (4)	C9—H9	0.9300
O5—H52	0.91 (2)	C10—H10	0.9300
N1—C1	1.342 (4)	C11—C10	1.377 (6)
N1—C5	1.342 (4)	C11—H11	0.9300
N2—C6	1.330 (5)	C12—C11	1.378 (6)
N2—H21	0.87 (2)	C12—C13	1.376 (5)
N2—H22	0.87 (2)	C12—H12	0.9300
C1—H1	0.9300		
			
O4^i^—Co1—O4	180.0	C2—C3—H3	120.6
O4^i^—Co1—N1	86.31 (10)	C4—C3—C2	118.7 (3)
O4—Co1—N1	93.69 (10)	C4—C3—H3	120.6
O4^i^—Co1—N1^i^	93.69 (10)	C3—C4—C5	119.3 (3)
O4—Co1—N1^i^	86.31 (10)	C3—C4—H4	120.3
O5^i^—Co1—O4^i^	91.75 (12)	C5—C4—H4	120.3
O5—Co1—O4^i^	88.25 (12)	N1—C5—C4	123.0 (3)
O5^i^—Co1—O4	88.25 (12)	N1—C5—H5	118.5
O5—Co1—O4	91.75 (12)	C4—C5—H5	118.5
O5^i^—Co1—O5	180.0	O1—C6—N2	123.5 (3)
O5^i^—Co1—N1	92.59 (11)	O1—C6—C2	119.1 (3)
O5—Co1—N1	87.41 (11)	N2—C6—C2	117.3 (3)
O5^i^—Co1—N1^i^	87.41 (11)	O2—C7—O3	124.3 (3)
O5—Co1—N1^i^	92.59 (11)	O2—C7—C8	119.3 (3)
N1—Co1—N1^i^	180.000 (1)	O3—C7—C8	116.3 (3)
Co1—O4—H41	124 (3)	C9—C8—C7	119.6 (3)
Co1—O4—H42	101 (4)	C13—C8—C7	123.9 (3)
H41—O4—H42	106 (3)	C13—C8—C9	116.5 (3)
Co1—O5—H51	136 (3)	C8—C9—H9	119.2
Co1—O5—H52	116 (3)	C10—C9—C8	121.5 (3)
H51—O5—H52	107 (3)	C10—C9—H9	119.2
C1—N1—Co1	121.3 (2)	C9—C10—H10	120.1
C5—N1—C1	117.3 (3)	C11—C10—C9	119.7 (4)
C5—N1—Co1	121.1 (2)	C11—C10—H10	120.1
C6—N2—H21	118 (3)	C10—C11—C12	120.4 (4)
C6—N2—H22	122 (3)	C10—C11—H11	119.8
H21—N2—H22	118 (4)	C12—C11—H11	119.8
N1—C1—C2	123.3 (3)	C11—C12—H12	120.6
N1—C1—H1	118.3	C13—C12—C11	118.8 (4)
C2—C1—H1	118.3	C13—C12—H12	120.6
C1—C2—C3	118.4 (3)	F1—C13—C12	117.1 (3)
C1—C2—C6	122.5 (3)	F1—C13—C8	119.8 (3)
C3—C2—C6	119.1 (3)	C12—C13—C8	123.0 (4)
			
O4^i^—Co1—N1—C1	−51.9 (3)	O1—C6—C2—C3	−19.9 (5)
O4—Co1—N1—C1	128.1 (3)	N2—C6—C2—C1	−20.0 (5)
O4^i^—Co1—N1—C5	135.3 (3)	N2—C6—C2—C3	161.2 (3)
O4—Co1—N1—C5	−44.7 (3)	O2—C7—C8—C9	−149.0 (3)
O5^i^—Co1—N1—C1	39.7 (3)	O2—C7—C8—C13	30.0 (5)
O5—Co1—N1—C1	−140.3 (3)	O3—C7—C8—C9	29.2 (4)
O5^i^—Co1—N1—C5	−133.1 (3)	O3—C7—C8—C13	−151.7 (3)
O5—Co1—N1—C5	46.9 (3)	C7—C8—C9—C10	178.3 (3)
Co1—N1—C1—C2	−172.2 (3)	C13—C8—C9—C10	−0.8 (5)
C5—N1—C1—C2	0.9 (5)	C7—C8—C13—F1	4.5 (5)
Co1—N1—C5—C4	172.4 (3)	C7—C8—C13—C12	−178.5 (4)
C1—N1—C5—C4	−0.8 (5)	C9—C8—C13—F1	−176.4 (3)
C3—C2—C1—N1	−0.8 (5)	C9—C8—C13—C12	0.5 (6)
C6—C2—C1—N1	−179.5 (3)	C8—C9—C10—C11	1.1 (6)
C1—C2—C3—C4	0.5 (5)	C12—C11—C10—C9	−1.2 (7)
C6—C2—C3—C4	179.3 (3)	C11—C12—C13—F1	176.4 (4)
C5—C4—C3—C2	−0.4 (5)	C11—C12—C13—C8	−0.6 (7)
N1—C5—C4—C3	0.5 (6)	C13—C12—C11—C10	1.0 (7)
O1—C6—C2—C1	158.9 (4)		

Symmetry codes: (i) −*x*+1, −*y*+1, −*z*+1.

### Hydrogen-bond geometry (Å, °)

**Table:**

*D*—H···*A*	*D*—H	H···*A*	*D*···*A*	*D*—H···*A*
N2—H21···O1^ii^	0.87 (2)	2.04 (3)	2.902 (5)	171 (4)
N2—H22···F1^iii^	0.87 (2)	2.54 (4)	2.916 (5)	107 (2)
N2—H22···O2^iii^	0.87 (2)	2.26 (3)	3.116 (5)	172 (4)
O4—H41···O3	0.91 (5)	2.06 (4)	2.885 (4)	151 (4)
O4—H42···O3^iv^	0.90 (3)	1.86 (5)	2.761 (4)	178 (5)
O5—H51···O2^v^	0.90 (4)	1.80 (4)	2.695 (4)	172 (4)
O5—H52···O3^vi^	0.91 (2)	1.95 (4)	2.798 (4)	156 (4)

Symmetry codes: (ii) −*x*, −*y*+1, −*z*+2; (iii) *x*−1, *y*, *z*; (iv) −*x*+1, −*y*+2, −*z*+1; (v) −*x*+2, −*y*+1, −*z*+1; (vi) *x*, *y*−1, *z*.
